# Mass and Mobility of Ions Produced by Radioactive Sources and Corona Discharges

**DOI:** 10.1021/acs.analchem.4c01796

**Published:** 2024-08-26

**Authors:** Fabian Schmidt-Ott, Anne Maisser, George Biskos

**Affiliations:** † Climate and Atmosphere Research Centre, The Cyprus Institute, Nicosia 2121, Cyprus; ‡ Institute for Atmospheric and Earth System Research, University of Helsinki, Helsinki 00014, Finland; § Faculty of Civil Engineering and Geosciences, Delft University of Technology, Delft CN 2628, The Netherlands

## Abstract

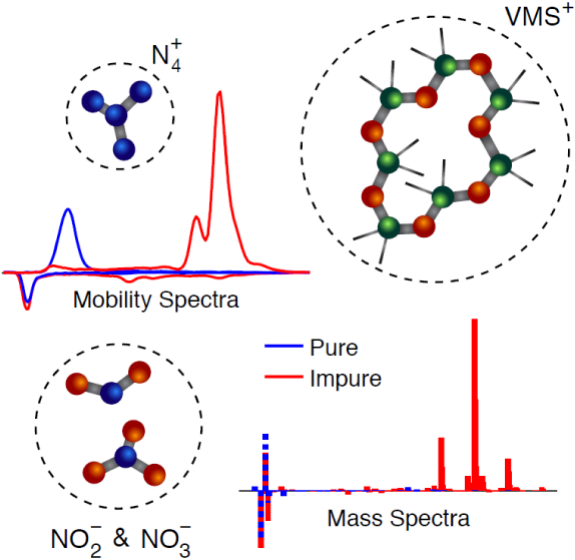

Positive and negative ions produced by radioactive sources and corona discharges in gases find a number of applications, including charging aerosol particles prior to their measurement by electrical and/or electrical mobility techniques. The degree to which these ions can charge aerosol particles depends on their mobility and mass; properties that are strongly affected by the composition of the carrier gas and the impurities that it contains. We show that when the purity of the carrier gas is increased, the mobility of both positive and negative ions increases by more than 50%, whereas the respective masses reduce by more than 50%. In most cases, the dominant positive species is N_4_^+^, whereas NO_2_^–^ and NO_3_^–^ prevail for the negative polarity. Differences in ion mobility and mass resulting from the two ionization methods (i.e., radioactive source and corona discharges) remain limited. When volatile methyl siloxanes (VMS) are introduced deliberately to the gas, the mobility of the cations decreases by 39% and their mass increases by 385%, while the dominant mobility and mass peaks of the negative ions remains almost unaffected. Interestingly, introduction of VMS also leads to consistent and reproducible positive ion properties across all variations of the experiments, which can be especially relevant for charging aerosol particles in a reproducible manner. Taken together, the new measurements we report in this paper corroborate prior knowledge that the composition and purity of the carrier gas strongly influence the properties of positive and negative ions generated in aerosol neutralizers, and provide new evidence regarding their evolution in the presence of impurities.

## 1 Introduction

The realization that gases can be ionized by different processes has attracted the interest of a number of researchers since the late 19th century.^1−3^ In the early years, properties of ions produced in gases were systematically studied using drift tubes, where their mobility could be determined from the time they required to traverse the length of the tube when pulled by an electrostatic field.^4−6^ Mass measurements of charged particles began in the effort to determine the mass-to-charge ratio of electrons produced from cathode rays by J. J. Thomson in the late 1890s.^7^ Only in the 1940s, measurement of ions having a wide range of masses was enabled by the development of the time-of-flight method, which is still widely used for chemical identification in various disciplines.^8,9^ These advancements have greatly expanded our understanding of ionized gases, and the ability to use them effectively for a number of applications.

Currently, ionized gases are readily used to charge aerosol particles prior to characterization by electrical (i.e., with an electrometer) or electrical mobility techniques. Electrical mobility classification of aerosol particles, commonly achieved by a differential mobility analyzer (DMA), is one of the most effective methods for sizing aerosol particles having diameters that range from those of clusters, comprised of a few atoms, to nanoparticles of several hundred nanometers. Both the differential mobility particle spectrometer (DMPS)^10^ and the scanning mobility particle sizer (SMPS)^11^ combine a DMA with a particle counter to determine the mobility/size distributions of aerosol particles. Since the first introduction of the DMA, efforts have been made to push the sizing of particles in the sub-5 nm range by shortening the classification section and using high sheath flows.^12−14^ Recent efforts in high-flow DMAs have shown that a parallel plate configuration (pp-DMA) is advantageous for the measurement of atomic and molecular clusters as it can achieve high transmission and resolving power in the sub-4 nm size range.^15,16^

As reflected by the discussion above, DMAs are readily used for characterizing aerosol particles in different contexts. Prior to measuring their electrical mobility with a DMA, however, aerosol particles need to be charged. A highly robust and widely employed method to achieve this is diffusion charging, where the particles are exposed to an ionized gas and are consequently charged by the attachment of gas-suspended ions on their surface. The ion-to-particle attachment probability primarily depends on the ionic mass and mobility, as described theoretically by Fuchs.^23^

Employing Fuchs’s model, Tigges et al.^24^ carried out a sensitivity analysis to determine the importance of the ion properties (specifically their electrical mobility) on the charging probability. Using ion mobility ranges reported in the literature ( = 1.10–1.65 cm^2^/Vs and  = 1.15–2.09 cm^2^/Vs) and the Kilpatrick^25^ model that relates the mobility to the mass of ions, they showed that the fraction of charged particles undergoing bipolar diffusion charging can deviate up to several tens of percent compared to the predictions using commonly assumed ion properties. Their results highlight the importance of having accurate values of the mobility and mass of the charging ions for predicting the charge distribution of the particles undergoing diffusion charging, which consequently affects the sizing and counting of aerosol size spectrometers that employ diffusion chargers.

Ions in gases can be produced by a range of sources, including radioactive materials, corona discharges, and X-rays. In any of these cases, the molecules of the gas (i.e., O_2_ and N_2_ in the case of air) are ionized, and the resulting ions subsequently react with less abundant trace species to form more stable ions; i.e., ions less likely to evolve further.^26^ The evolution of the ionic species in different gaseous environments still remains an open question. Dominant ionic species of negative polarity that have been observed to form in air include NO_3_^–^, HNO_3_^–^, HCO_3_^–^, HNO_3_^–^, NO_2_·(H_2_O)_*n*_^–^, NO_3_·(H_2_O)_*n*_^–^, HNO_3_·(H_2_O)_*n*_^–^, and NO_3_(HNO_3_)_*m*_·(H_2_O)_*n*_^–^.^26−28^ For the positive ions produced in air, the most abundant species are more limited, including (H_3_O)_*m*_·(H_2_O)_*n*_^+^, NH_4_^+^, and (H_2_O)_*n*_^+^.^26,29−31^ This variety reflects the complexity in the formation of ions, and in part explains the variabilities in measurements reported in the literature where different experimental setups and procedures are employed.

The nature of charger ions is difficult to determine due to the complexity of the required instrumentation and the sensitivity of the ionic species in trace amounts of impurities present in the carrier gas. Impurities in the gas play a dominant role in the formation of ions from radioactive, X-rays, and corona chargers. Steiner and Reischl^32^ have shown that the composition of trace impurities in highly pure gases can vary from one experimental setup to another. This is in part because trace species, such as siloxanes and phthalates, which outgas from any plastic parts employed in the experimental setups, including o-rings, ferules, and flexible tubes, are difficult to avoid.^17−36^ Other studies have shown that the mobility spectrum of these ions is only weakly affected by the bulk carrier gas itself (i.e., nitrogen or synthetic air).^33,37,38^

In addition to the impurities present in the carrier gas, the charging method employed can also influence the properties of ions and consequently affect the aerosol charge distribution.^39,40^ Results from previous studies investigating the extent to which the charging method influences the ion composition are, however, somewhat ambiguous. Kallinger et al.^41^ measured the mobility of ions generated by various charging methods, including bipolar corona discharges, X-rays, and radioactive ionization. Their results demonstrate that the negative ions produced by different sources exhibit a wide range of mobilities, ranging from 1.68 to 2.09 cm^2^/Vs. On the other hand, the electrical mobility of positive ions remains rather unaffected, exhibiting values of ca. 1.55 cm^2^/Vs for all ionization methods. In contrast, Tauber et al.^38^ measured both the mass and electrical mobility of ions generated by a radioactive source and a plasma charger, but did not observe any differences in the properties of either the anions or the cations related to the charging method used.

Each of the previously mentioned studies has focused on one or two parameters that influence the composition of the ions produced from different sources (i.e., the bulk composition of the carrier gas; the impurities that the carrier gas contains; or the type of ion source employed), oftentimes providing data that are difficult to compare, and subsequently used to draw generalized conclusions. What is more, a large number of these studies reports measurements of ions of only one polarity. To the best of our knowledge, simultaneous measurement of the mass and mobility of ions of both polarities produced by different sources, considering all the above-mentioned parameters have not been reported thus far.

To address this gap, we have carried out systematic measurements using a mobility analyzer and a mass spectrometer in order to determine how the properties of positive and negative ions produced by a radioactive source and a corona discharge can be affected by (1) the purity of the carrier gas, (2) the bulk composition of the carrier gas, (3) the type of ion source, and (4) the use of silicone-based conductive tubing in sampling lines. The rest of the paper is organized as follows: Section 2 provides details of the experimental setup versions and procedures used for each measurement, Section 3 discusses the results, and Section 4 provides a summary of the most important conclusions.

## 2 Experimental Section

Two aerosol charge neutralizers were employed in our experiments: a ^241^Am-source radioactive neutralizer (RN; Model 5622, GRIMM Aerosol Technik GmbH, Germany) and a bipolar corona neutralizer (CN; Model 1090, MSP Corp., Minnesota, USA). ^241^Am produces alpha radiation upon its decay, which subsequently ionizes the overlaying gas. The CN produces a bipolar charge spectrum by means of a corona discharge formed at the tip of a needle set at an alternating current. In all the experiments, a high enough gas flow rate of 7.5 L per minute (lpm) was passed through the charge neutralizers to minimize the losses for small, highly mobile ions. We should note here that the ^241^Am radioactive neutralizer was newly purchased and not used for any measurements prior to this study, whereas the CN had been employed only in a few lab experiments before the tests were carried out.

The mobility of the ions produced by the two neutralizers was measured using a high-resolution, high-transmission pp-DMA (SEADM P5 DMA) coupled to a Faraday cage electrometer (FCE; SEADM). The pp-DMA was operated with a resolving power—defined as the peak DMA voltage over the full width at half-maximum of the same peak (V/FWHM)—of 65, as this was determined using tetraheptylammonium (THA^+^) monomer for the calibration (cf. Figure S1). We should note here that the pp-DMA has a significantly higher transmission (∼50% for THA^+^) compared to conventional cylindrical DMAs, making it highly suitable for measuring light and highly mobile ions and nanoparticles having sizes up to ca. 4 nm.^13,42^ In all the experiments reported in this work, the voltage of the pp-DMA was stepwise increased to select ions having mobilities that range between 0.8 and 2.8 cm^2^/Vs, with steps of ca. 0.01 cm^2^/Vs.

The mass of the ions produced by the two charge neutralizers was measured with a custom-made atmospheric pressure interface time-of-flight mass spectrometer (API-TOF-MS).^43^ The API-TOF-MS samples ions from atmospheric pressure, which are subsequently focused and selected by an aerolens, ion funnels, and multipoles, before they are separated according to their mass-to-charge ratio in the TOF chamber.^44^ The masses of the ions that the API-TOF-MS can measure range from 28 to 12,000 Da. As shown by preliminary measurements, the transmission of the instrument is approximately 1% at 410 Da (corresponding to THA^+^), the mass resolving power at 250 Da is 15,000, and the mass accuracy is ca. 10 ppm.^43^

**Figure 1:**
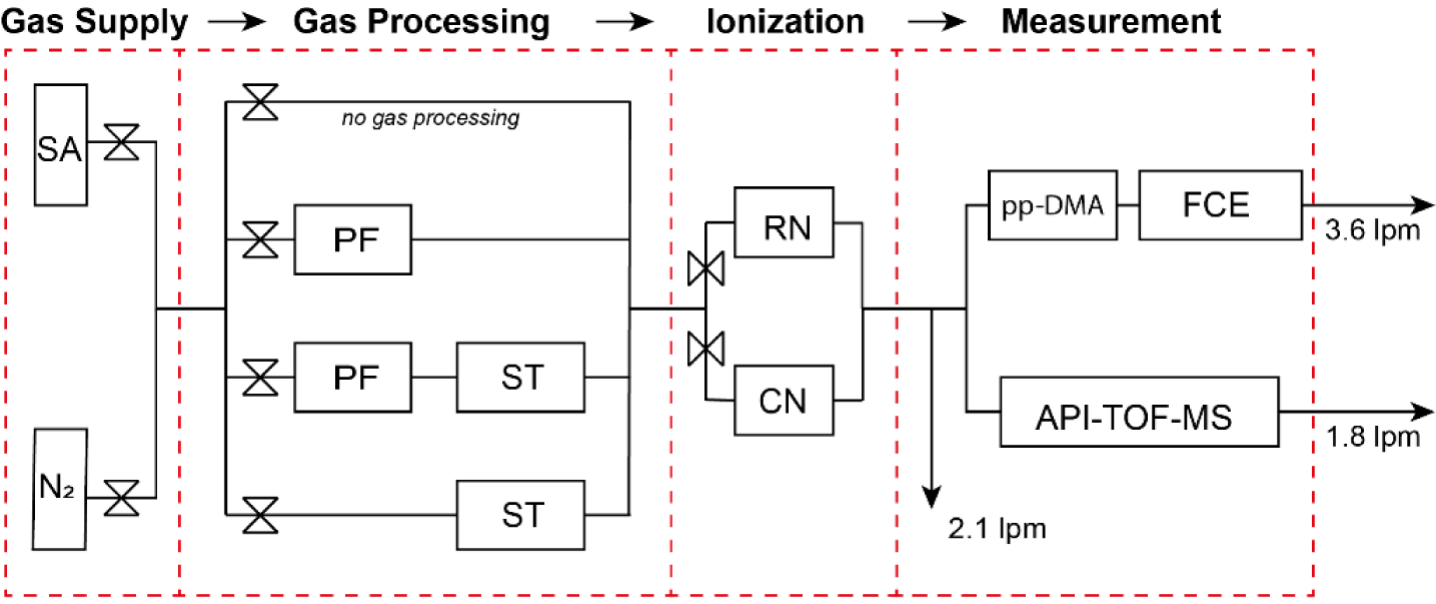
Schematic layout of the experimental setup showing the different stages of our system, including the gas supply, the gas processing, the ionization, and the measurement stages. In each stage only one of the flow pathways (illustrated with conceptual valves) was open. All measurements were carried out with the DMA and the API-TOF-MS operating in parallel. Key: SA, synthetic air; N_2_, nitrogen; PF, purifier; ST, silicone tubing; RN, radioactive neutralizer; CN, corona neutralizer; pp-DMA, parallel-plate differential mobility analyzer; API-TOF-MS, atmospheric pressure interface time-of-flight mass spectrometer; FCE, faraday cage electrometer.

The experimental setup for the measurements reported in this paper is shown in Figure 1. In brief, high-purity gases were first passed through a processing stage, where they were further purified and/or deliberately contaminated by adding a small piece (1 cm long and 0.6 cm in internal diameter) of silicone-based tube (TSI, ID 3001788, USA) upstream the charge neutralizers. The silicone tube had been newly purchased and not used in other experimental setups prior to this study. The gases used in our measurements were either nitrogen or synthetic air (99.999% purity). According to the provider of the gases, trace species in the nitrogen bottle were <3 ppm for O_2_, <5 ppm for H_2_O, and <0.2 ppm for organic compounds. In some of the experiments we used a purifier (Agilent, Model CP17973) to further remove impurities from the nitrogen gas. As specified by the manufacturer, the concentrations of impurities downstream the purifier are <50 ppb for O_2_, <0.1 ppm for H_2_O, and <0.1 ppm for organic compounds.

We took special care to minimize the sources of impurities in our experimental setup. All the tubing in the system consisted of stainless steel, except for the experiments in which we added the silicone-based tube, while valves were deliberately omitted to prevent contamination from grease. Furthermore, the DMA was cleaned regularly and the tip of the corona needle was kept as free from impurities as possible by scrubbing it with a piece cloth soaked in methanol. Before each measurement, the carrier gas was passed through the system for an extended period (up to 5 h) to ensure the maximum removal of any impurities adhering to the inner walls. Possible impurities from the DMA sheath flow blower were minimized by maintaining it at room temperature.

In most of the experiments, the pp-DMA and API-TOF-MS were used in parallel. Although the in-series setup can provide rich information for the studied species (cf. Figure S2), the parallel configuration exhibits important advantages for the study of charger ion properties. First, the parallel configuration allowed the detection of a wider range of ion masses, down to 28 Da. This is due to smaller distances and hence lower ion transmission losses between ion production and detection, which is important for the low-mass/high-mobility species. Another reason for using the parallel configuration was to avoid charge exchange collisions within the DMA. In these collisions, a neutral molecule can combine with an ion or take up its charge, changing the mass spectrum.^34^ Implementing a parallel configuration for mass and mobility measurement ensures that those charge exchange reactions do not impair the mass measurement.

## 3 Results and Discussion

In this section, we discuss the mass and mobility spectra of ions produced by the two bipolar chargers using different paths in our experimental setup, as shown in Figure 1. The section is divided in four subsections, focusing on the effect that different experimental configurations and parameters have on the mobility and mass spectra: (1) gas purification, (2) carrier gas, (3) ionization method, and (4) the use of silicone tubing as a means of introducing impurities. In each section, we compare the results obtained between the two configurations, with one of the two serving as the basis for comparison. In all figures, the electrical mobility values are expressed as the reduced electrical mobility (*Z*_0_) determined by
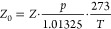
1where *Z* is the measured mobility at temperature *T* (K) and pressure *p* (bar) in the classification zone of the DMA. All electrical mobility equivalent diameters provided in this work are calculated according to the Stokes–Millikan formalism, linking the measured mobility to an effective cluster/particle mobility diameter.^45,46^

In all the spectra provided along the discussion, positive and negative ions are represented, respectively, on the positive and negative sides of the *y*-axis. The masses of ions corresponding to specific species in the API-TOF-MS spectra are summarized in Tables S1 and S2.

### 3.1 Effect of Gas Purification

**Figure 2:**
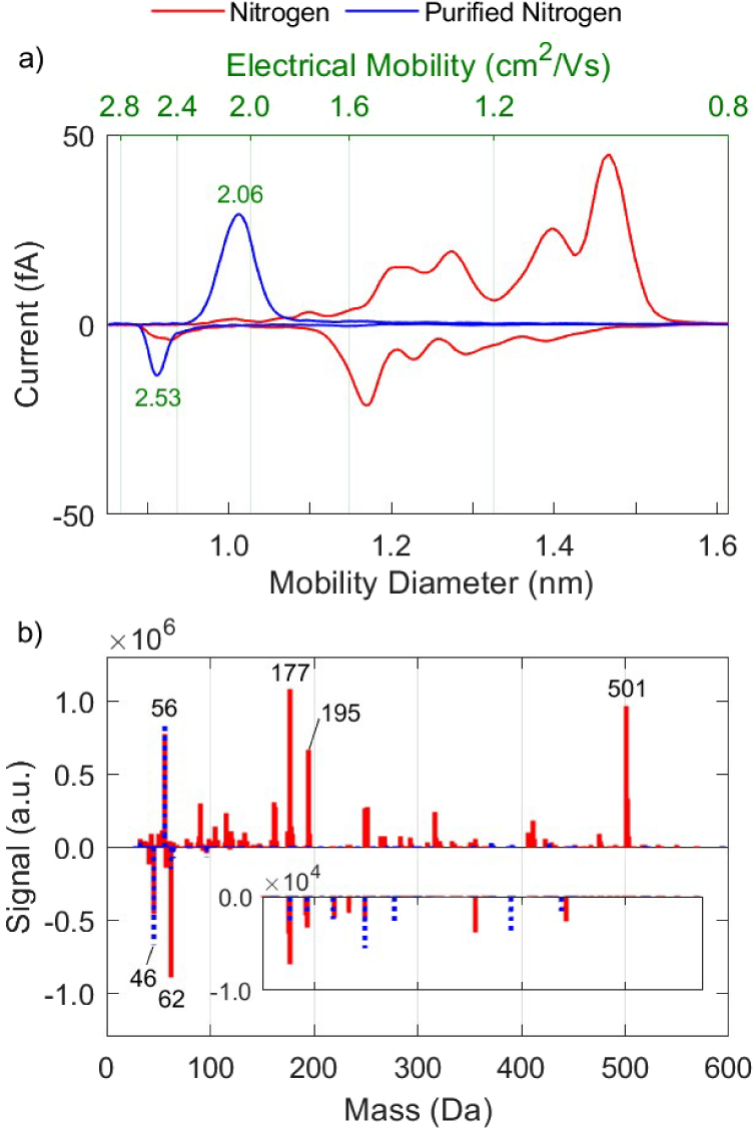
Mobility (a) and mass (b) spectra of positive and negative ions produced by the RN in nitrogen and purified nitrogen.

As a first set of measurements, we used N_2_ directly from the bottle, or after purification—to the best degree possible—by passing it through a purifying system that effectively removes O_2_, H_2_O, and hydrocarbons as described in the previous section. As shown in Figure 2, ions produced in nitrogen without the purification step have mobilities that range widely from ca. 0.9 to 2.5 cm^2^/Vs, and dominant masses of up to 501 Da. The mean mobilities of the ions produced in nitrogen are  1.2 cm^2^/Vs and  1.6 cm^2^/Vs, whereas the mean masses are  219 Da and  149 Da. When the nitrogen from the bottle is purified, both the mobility and mass spectra change significantly. Specifically,  and  increase by 65% and 52%, whereas  and  decrease by 55% and 61%, respectively, due to the removal of the high-mass/low-mobility organic species. Given that the purifier was placed directly downstream the gas bottle, the organic species likely originate from the gas bottle.

The most abundant negative species exhibit masses at 46 and 62 Da, corresponding to the nitrite (NO_2_^–^) and nitrate (NO_3_^–^) ions, respectively, that play a key role in the evolution of the rest of the anions. These highly electronegative species act as electron scavengers, thereby inhibiting the negative ionization of compounds with a lower electron affinity.^34,47^ As a result, species that are less electronegative compared to NO_3_ remain either neutral or become positively charged. The larger variety of positively charged species, as opposed to negative species, can largely be explained through this process.

The mobility peak of both NO_2_^–^ and NO_3_^–^ ions combined is at 2.53 cm^2^/Vs, as illustrated in Figure 2a, but can include species such as O^–^ or OH^–^ that may be present and classified by the DMA but not captured in the API-TOF-MS spectra, as they fall below its lower detection threshold of 28 Da. Despite the low concentration of O_2_ in the purified nitrogen gas, evidently it still plays an important role in the evolution of anions, contributing to the formation of NO_2_^–^ and NO_3_^–^. We should note that even species (impurities) present in concentrations at the ppb level in the carrier gas can play a significant role in the formation of ions, as they correspond to concentrations of ca. 10^10^ molecules/cm^3^.

Positive ions produced in both purified and nonpurified nitrogen have a dominant peak in the API-TOF-MS spectra at a mass of 56 Da. The measurement accuracy of the mass spectrometer shows in fact that the mass is 56.013 Da. This high accuracy (36 ppm, corresponding to an uncertainty of ±0.002 Da for this mass), together with the measured isotopic ratios (Signal_57 Da_/Signal_56 Da_ = 0.0152 ± 0.0016), strongly indicate that this peak corresponds to N_4_^+^. Garcia et al.^48^ showed that the disassociation energy of N_4_^+^ is ca. 0.87 eV/mol, and thus it can be in equilibrium with ionized nitrogen: . This is further supported by drift tube measurements reported by Varney^49^ and Saporoschenko,^50^ who demonstrated that the formation rate of N_4_^+^ from N_2_ and N_2_^+^ is higher than the dissociation rate into N_2_^+^ and N_2_ at ca. 1.3 mbar. They also showed that the equilibrium constant for N_4_^+^/ N_2_^+^ rises proportionally with pressure, so N_4_^+^ should be strongly dominant with respect to N_2_^+^ at 1 bar. It is therefore not surprising that N_4_^+^ is formed when nitrogen at atmospheric pressure is used as a carrier gas in our measurements.

The above explanation indicates that N_2_^+^ is formed by sources similar to those we investigate here, suggesting that the ionization of N_2_ and the subsequent charge transfer from N_2_^+^ to surrounding species is one of the key charging pathways, if not the main, in the evolution of the ions. Considering the dominance of N_2_ (99.999%), this pathway is not surprising, and to our knowledge, it has not been demonstrated experimentally yet. We should note here that the transmission of the mass spectrometer at 56 Da, corresponding to N_4_^+^, represents only a minor fraction, approximately 0.01, compared to transmission at masses above 200 Da. Considering that, we can conclude that N_4_^+^ is the most dominant species in the positive polarity, and consequently the ion pathway involving the formation of N_2_^+^ is highly likely. Further experimental evidence, with mass spectra going down to less than 28 Da would be required for further supporting that.

### 3.2 Effect of the Carrier Gas

**Figure 3:**
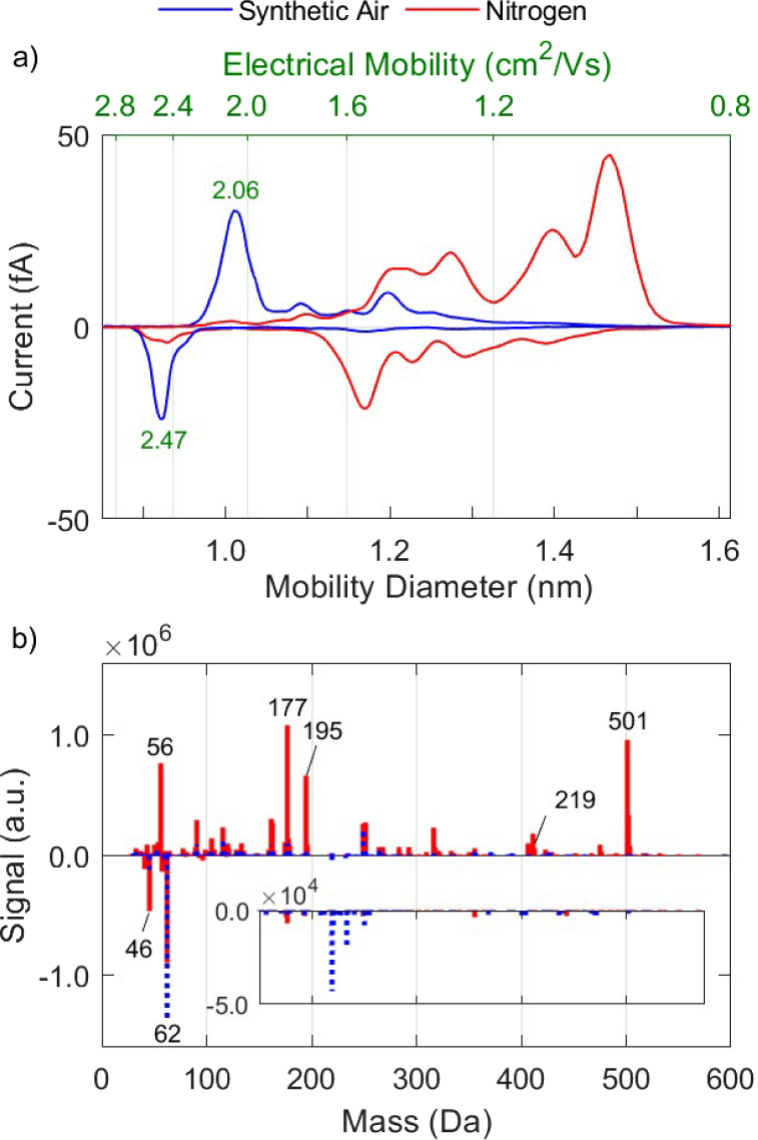
Mobility (a) and mass (b) spectra of positive and negative ions produced by the RN in synthetic air and nitrogen.

Changing the carrier gas from nitrogen to synthetic air increased  and  by 52% and 47% (i.e., from 1.2 to 1.9 cm^2^/Vs and from 1.6 to 2.4 cm^2^/Vs), respectively, as shown in Figure 3. The respective values of  and  decreased by 17% and 39% (i.e., from 219 to 181 Da and from 149 to 91 Da). The results reported here are in contrast to previous reports by Liu et al.^33^ and Steiner et al.^37^, who showed that the mean values of both the ion mass and mobility produced in nitrogen and synthetic air are comparable. The difference in mean ion mass and mobility resulting from the choice of the carrier gas in our experiments can be attributed to different levels of contamination originating from the respective gas containers, which in both cases correspond to organic species (cf. Table S1).

When using synthetic air as the carrier gas, the mobility spectra exhibit dominant peaks at 2.06 cm^2^/Vs for the positively charged ions and 2.47 cm^2^/Vs for the negatively charged ions, as shown in Figure 3. While the negative mobility peak likely corresponds to NO_2_^–^, NO_3_^–^, and smaller ions, it is unclear which species comprise the positive mobility peak. The high mobility peak at positive polarity may be due to cluster ions, containing species such as H_2_O, which are in a dynamic equilibrium with other gas molecules.^51^ Those cluster ions are weakly bound and fragment easily in the mass spectrometer.

It is worth pointing out the absence of the N_4_^+^ peak in the mass spectrum when synthetic air is employed as the carrier gas (cf. discussion in Section 3.1). A possible explanation is that oxygen, which is much more abundant in synthetic air than in nitrogen, consumes all the N_2_^+^,^52^ limiting the formation of N_4_^+^ through the reaction pathway discussed in Section 3.1. Similar results were reported in one of the first drift tube measurements reported by Luhr,^5^ where N_4_^+^ ions were produced by glow discharge in pure nitrogen, but their concentration strongly diminished upon introduction of oxygen to the system. This shows that for laboratory settings, where nitrogen is frequently used as a carrier gas, N_4_^+^ comprises a dominant charge carrier, but this is not the case when passing air (either synthetic or ambient) through the neutralizers.

### 3.3 Effect of the Ionization Source

**Figure 4:**
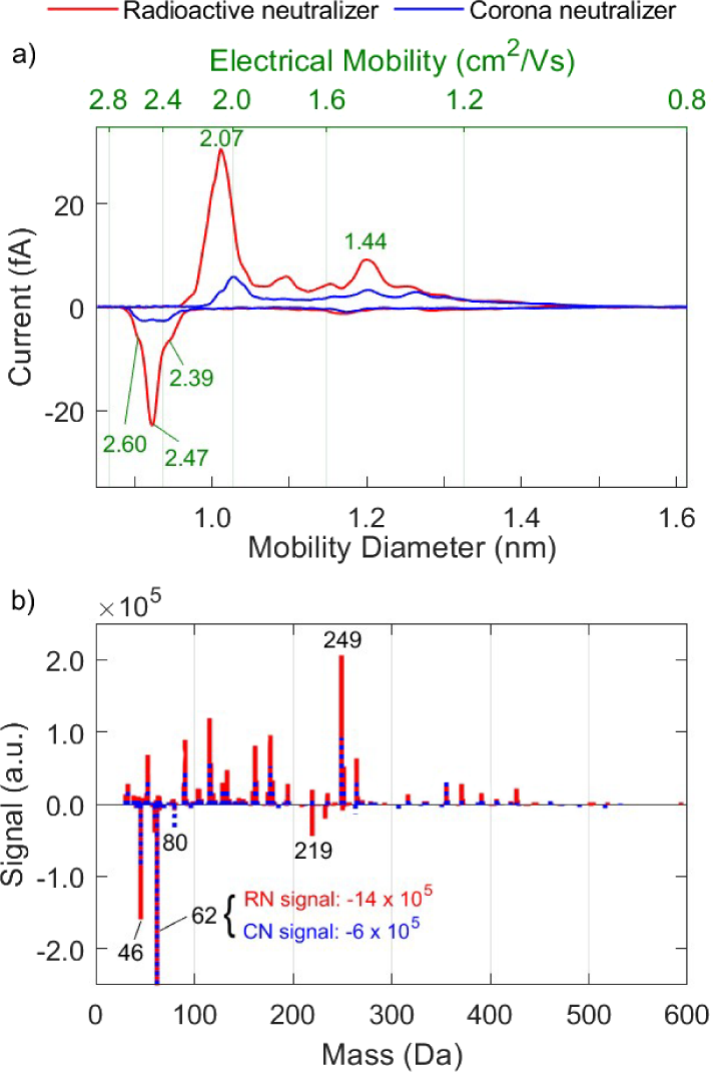
Mobility (a) and mass (b) spectra of positive and negative ions produced by the RN and CN in synthetic air.

Figure 4 compares the mobility and mass spectra of ions produced by the radioactive and the corona neutralizer in synthetic air. The mobilities of ions produced by the two neutralizers agree well with each other, with  and  deviating by 10% and 6%, and  and  by 3% (cf. Table 2), which can be considered within the uncertainty levels of our experiments.

Similarities in the mobility of positive ions among different neutralizers were also reported by Kallinger et al.,^41^ who compared the mobilities of ions produced by a custom-built ^241^Am-source and the corona neutralizer. According to their measurements, the mean mobilities of positive ions produced in synthetic air by both neutralizers differed by only 4.4%, whereas a larger deviation (11.3%) was reported for the negative ions. Along the same lines, Tauber et al.^38^ investigated the mobility and mass of ions produced by a bipolar plasma or an ^241^Am source, and reported that they exhibited similar mass spectra in the negative polarity, but not in the positive polarity.

The concentration of ions produced by the two neutralizers in our measurements differ by a factor of 3–4 (cf. Table 2). This difference can be explained by the strong electric field in the CN, which leads to the deposition of ions before exiting the device. Differences in tubing length downstream the chargers (20 cm for CN and 12 cm for RN) can also contribute to those concentration differences, but their contribution is expected to be within less than 5%. Another cause for the low concentrations of ions produced by the CN may be associated with contaminants depositing on the tip of the corona needle, leading to a decrease in the production of ions over time.^17^

### 3.4 Effect of Conductive Silicone Tubing

**Figure 5:**
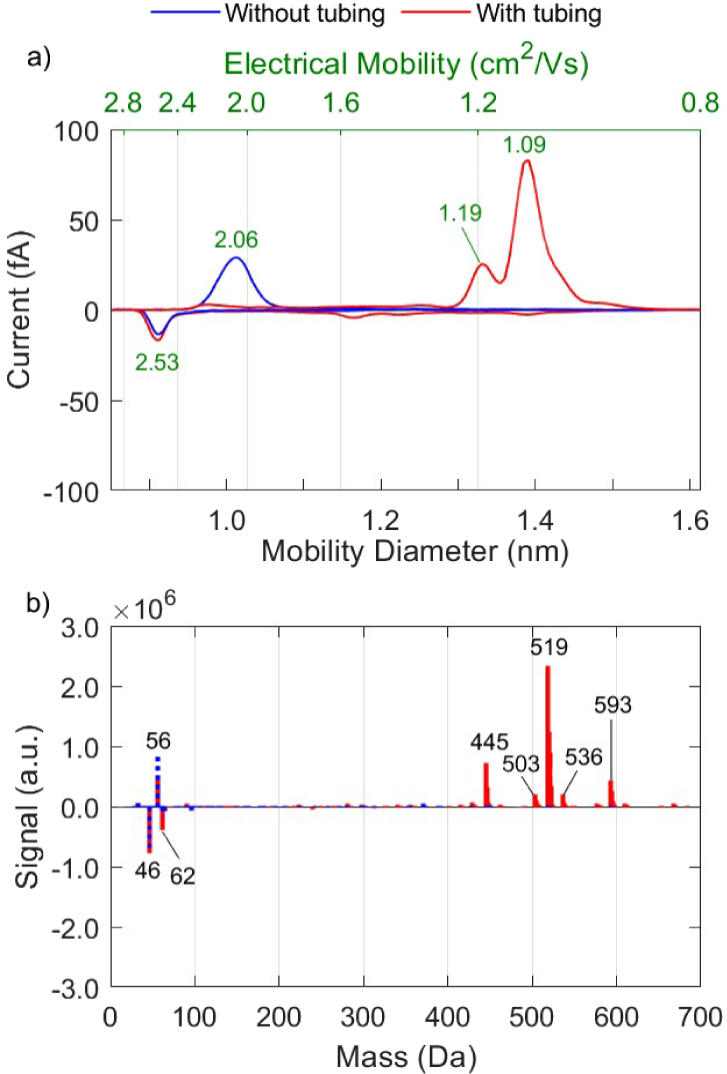
Mobility (a) and mass (b) spectra of positive and negative ions produced by the RN with and without a 1-cm-long piece of silicone tubing upstream the charger, using purified nitrogen as carrier gas.

As described in Section 2, we carried out a series of measurements using a 1-cm-long piece of silicone-based conductive tubing, which is typically used in sampling lines upstream aerosol mobility spectrometers that employ charge neutralizers similar to those investigated here. Figure 5 compares the ion mobility and mass spectrum from experiments where we used purified nitrogen as carrier gas, with or without conductive silicone tubing installed upstream the charger. Positively charged ions are strongly affected by the silicone tubing, as indicated by the appearance of dominant peaks at 1.19 and 1.09 cm^2^/Vs in the cation mobility spectrum. When adding the 1-cm-long piece of conductive tubing upstream the neutralizer,  and  decrease by 39% and 15%, and  and  increase by 385% and 76%, respectively.

The low mobilities of the positive ions that appear in high abundance can be allocated to siloxane clusters having the form [M + H–CH_4_]^+^_*n*_, [M + H]^+^_*n*_, or [M + NH_4_]^+^_*n*_, where *M* = C_2_H_6_SiO and *n* is the number of repeating units (cf. Table S-2). The most abundant siloxanes have masses of 445, 519, and 593 Da in the form of [M + H]^+^_*n*_ with *n* = 6, 7, or 8. These are cyclic siloxanes including methyl groups, and fall under the category of volatile methyl siloxanes (VMS), typically referred to as D6, D7, and D8 in the literature.^53^

**Figure 6:**
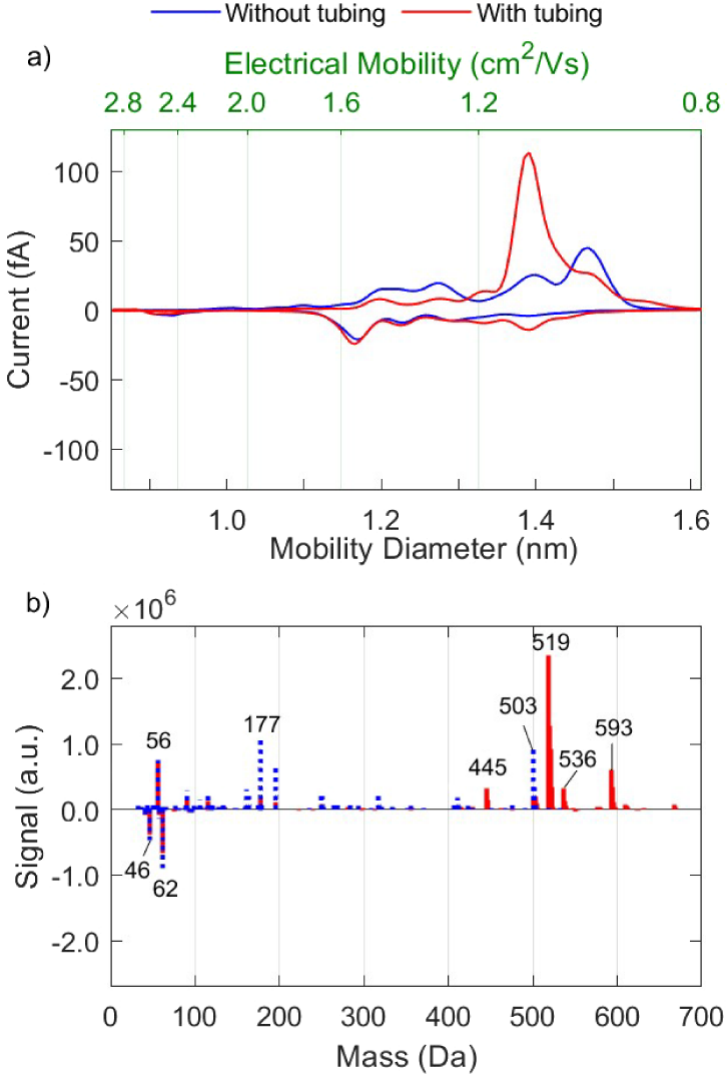
Mobility (a) and mass (b) spectra of positive and negative ions produced by the RN with and without a 1-cm-long piece of silicone tubing upstream the charger, using nonpurified nitrogen as carrier gas.

Considering that, the majority of VMS^+^ appearing in the spectra (Figure 5b) must have acquired their positive charge through proton capture, i.e., [M + H^+^], which can be explained by their high abundance and proton affinity. This charge-scavenging behavior of VMS is also reflected in the mobility spectra in Figure 5a, where the high-mobility peak at 2.06 cm^2^/Vs is replaced by low-mobility peaks that are associated with VMS^+^ species. Figure 6 shows that VMS even inhibit the charging of other organic impurities that are present, winning the competition for capturing the H^+^ and leading to heavy yet stable positive ions.

The high abundance of VMS^+^ ions in our measurements can be attributed to their high volatility, which leads to high outgassing rates from the tubing material, and thus to higher concentrations compared to other impurities in the carrier gas. Increasing the length of silicone-based tubing by a factor of 20, thus increasing the concentration of outgassed VMS in our system, had only a marginal impact on the measured VMS^+^ concentration, as shown in Figure S3, indicating that the formation of VMS^+^ is limited by the presence of species that can transfer protons.

Measurements conducted without silicone-based tubing exhibited significant variations in positive ion properties, with  and  ranging from 97.4 to 218.8 Da and 1.23 to 2.04 cm^2^/Vs, yielding differences within 125 and 66%, respectively (cf. Table 1). Interestingly, with the introduction of silicone-based tubing, these differences decreased to ca. 20% for both mass and mobility, demonstrating a stabilizing effect of VMS in the evolution of the ions produced by radioactive sources and corona discharges. This is advantageous as it can ensure repeatability in the properties of the resulting ionic species and consequently of any process they are involved in, including that of charge-neutralizing aerosol particles.

We should note here that the N_4_^+^ peak (56 Da) remained unaffected by the introduction of VMS into the system, indicating that the formation of VMS^+^ and N_4_^+^ are independent processes. This observation can be attributed to different charging mechanisms for each species: the removal of an electron for the formation of N_2_^+^, which leads to N_4_^+^, and the protonation for VMS^+^. Given that the two ionic species that we observe (VMS^+^ and N_4_^+^) are not in competition for acquiring their positive charge, explains why the N_4_^+^ peak is minimally influenced by the presence of VMS.

Table 1 shows the mean mass (), mean mobility ) and total concentration () of ions from the different paths used in our experimental setup. The values of  were obtained from the mobility distribution measurements. We should note here that due to the mass-dependent transmission and the fragmentation occurring in the mass spectrometer, the absolute values of the mean mass are primarily useful for qualitative assessments and comparisons.

**Table 1:**
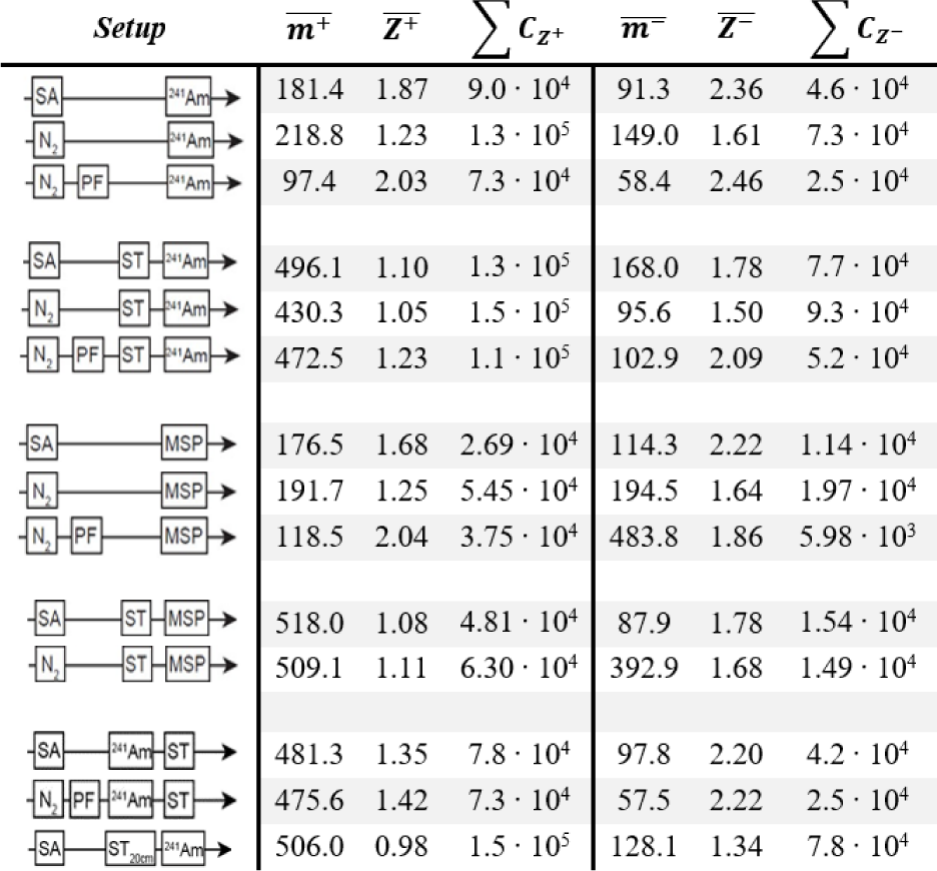
Summary of mean mass (Da), mean mobility (cm^2^/Vs) and total concentration (# cm^–3^) of ions measured with the different configurations of the experimental setup, in which the gas supply, gas processing, and ionization method were changed.

## 4 Conclusion

The measurements reported in this work demonstrate that the properties of ions produced by a radioactive source and a corona discharge are very similar, but they vary depending on the employed carrier gas (i.e., nitrogen or synthetic air) and the levels of impurities it contains. More specifically, our results show that the mean mobilities of the positive and negative ions ( and ) produced in nitrogen with the best possible purity level are 2.0 and 2.5 cm^2^/Vs, whereas their mean masses ( and ) are 97 and 58 Da, respectively. When the purity of the gas is decreased, the mean mobility and mass can vary by up to 65% and 385%, respectively, demonstrating that ion properties are highly sensitive to trace impurities present in the carrier gas from the bottle, or introduced at different points of the experimental setup.

Our measurements also show that electronegative species, such as NO_2_^–^ and NO_3_^–^, play an important role in the evolution of both positively and negatively charged ions. Their electrophilicity makes them effective electron scavengers, thereby reducing the probability of other neutral species from obtaining a negative charge, or of positive ions from combining/recombining with the electrons. This explains the low variability in the mass and mobility of negative ions we observe. When using nitrogen as carrier gas, these electrons are primarily released by the ionization of the N_2_, as suggested by our measurements. In turn, the resulting N_2_^+^ ions either grow to N_4_^+^ or transfer their positive charge to other species, including trace impurities in the carrier gas. Considering that the concentration and composition of the impurities can vary substantially from case to case, the mobility and mass of the positively charged species depend strongly on their type, explaining the high variability as indicated by our measurements and by reported results from different studies in the literature.

Using silicone-based conductive tubing upstream the charge neutralizers introduces impurities in the form of volatile methyl siloxane (VMS) species in the carrier gas. Even a short piece of conductive tubing (length of 1 cm) adds significant amounts of VMSs in the carrier gas, which pick up a good fraction of the initially formed positive charges to yield more stable ionic species. This observation suggests that VMSs intentionally introduced in the carrier gas (i.e., by deliberately using silicone-based conductive tubing in the sampling lines) can ensure low variability even for the positively charged ions, and thus high repeatability from one measurement to the other. Interestingly, the presence of VMS^+^ did not reduce the concentrations of N_4_^+^, indicating that these two species follow independent charging pathways: i.e., protonation for the formation of VMS^+^ and removal of electrons for N_4_^+^.

## Supporting Information Available

Calibration curve of tetraheptylammonium bromide (THABr) with a resolution of 65 (V_peak_/FWHM), mobility–mass resolved contour plot of negative ions produced by the RN in synthetic air, setup used for the DMA-MS in-series measurement, summary of identified ion masses, summary of identified siloxane species, and mobility and mass spectra of positive and negative ions produced by the RN in synthetic air using a silicone tubing length of 20 and 1 cm upstream the charger (PDF)
