# Supplementary material for: Mass and Mobility of Ions Produced by Radioactive Sources and Corona Discharges
Source: Anal Chem. 2024 Aug 26;96(36):14405–12. doi: 10.1021/acs.analchem.4c01796 (PMC11391406; doi:10.1021/acs.analchem.4c01796)
Supplement: Supplementary file 1 — ac4c01796_si_001.pdf [file ac4c01796_si_001.pdf]

Supplemental Information

Mass and Mobility of Ions Produced by Radioactive Sources and Corona Discharges

Fabian Schmidt-Ott [a,b] *, Anne Maisser [a], George Biskos [a,c]

[a] Climate and Atmosphere Research Centre, The Cyprus Institute, 2121 Nicosia, Cyprus

[b] Institute for Atmospheric and Earth System Research, University of Helsinki, 00014, Helsinki, Finland

[c] Faculty of Civil Engineering and Geosciences, Delft University of Technology, 2628 CN, Delft, The Netherlands

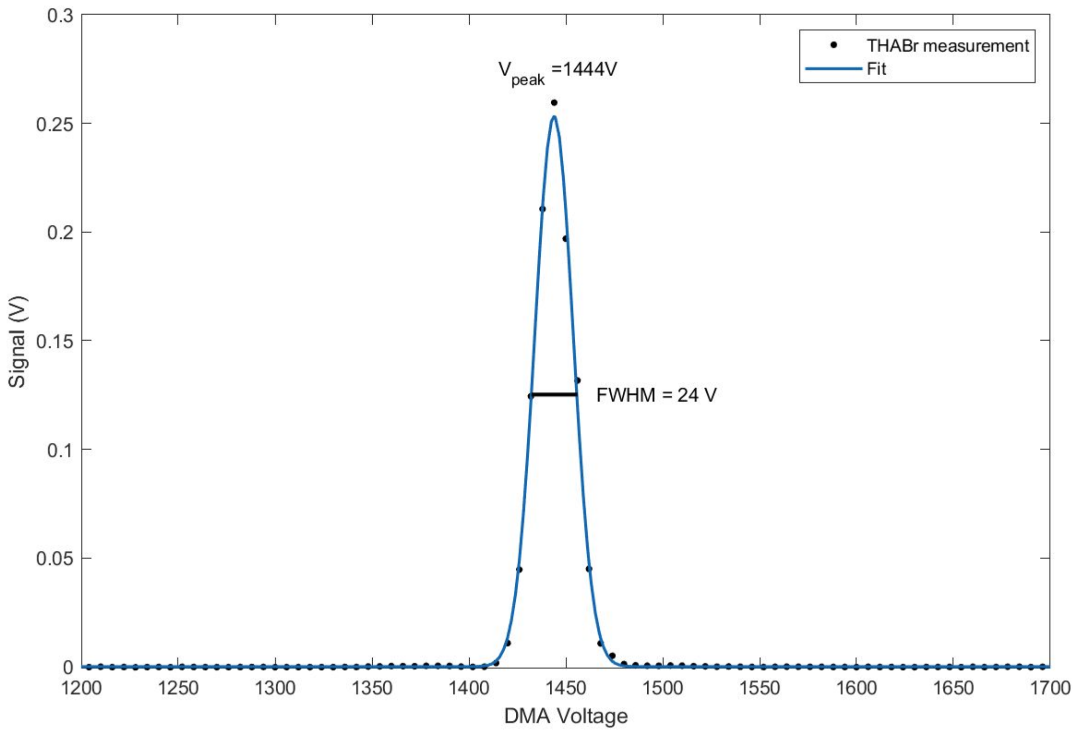

Figure S-1. Calibration curve of Tetraheptylammonium bromide (THABr) with a resolution of 65 (V_{peak}/FWHM).

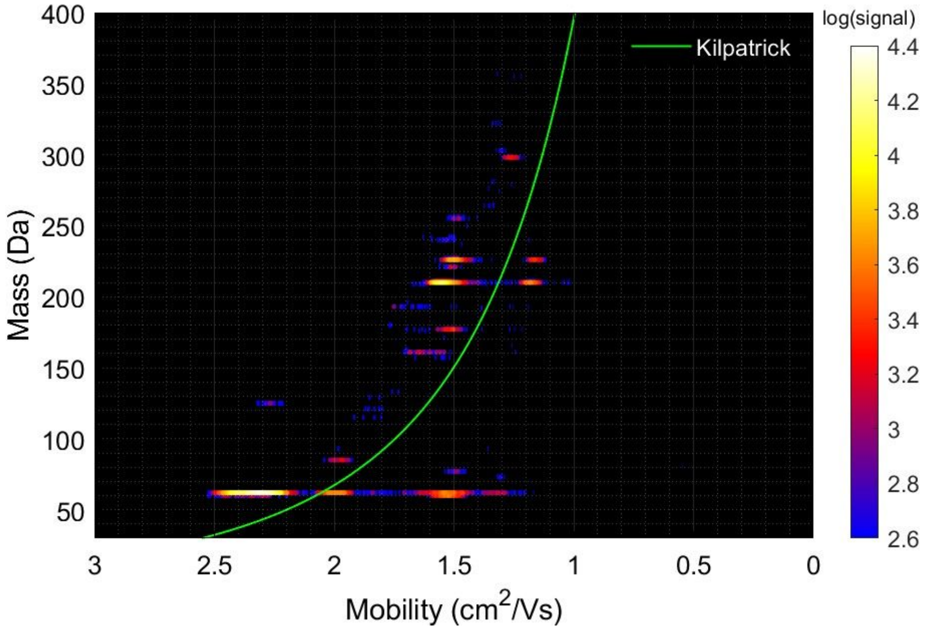

Figure S-2a. Mobility-mass resolved contour plot of negative ions produced by the RN in synthetic air.

Figure S-2b. Setup used for the DMA-MS in-series measurement.

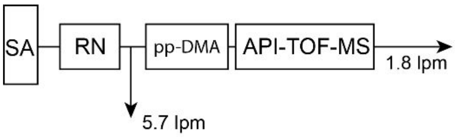

Table S-1. Summary of identified ion masses

Integer mass	Compound	Integer mass	Compound
28	N_2	88	C_3H_2
29	HN_2	89	C_7H_5
30	NO	90	C_7H_6
32	O_2	91	C_7H_7
36	C_3	103	C_8H_7
37	C_3H	107	C_7H_7O
38	C_3H_2	116	C_9H_8
39	C_3H_3	117	C_9H_9
41	CHN_2 & C_3H_5	118	C_9H_{10}
42	N_3 & CH_2N_2	119	C_8H_7O & C_9H_{11}
43	HN_3	121	C_8H_9O
46	NO_2	128	$C_{10}H_8$
50	C_4H_2	131	$C_{10}H_{11}$
51	C_4H_3	133	C_9H_9O
54	CN_3	135	$C_9H_{11}O$
56	N_4	147	$C_{11}H_{15}$
61	C_5H	159	$C_{12}H_{15}$

62	C_5H_2/NO_3	161	$C_{11}H_{13}O$
63	C_5H_3	163	$C_{10}H_{11}O_2$
65	C_5H_5	175	$C_{12}H_{15}O$
77	C_6H_5	177	$C_{11}H_{13}O_2$
78	C_6H_6	193	$C_{12}H_{17}O_2$
79	C_6H_7	195	$C_{11}H_{15}O_3$

Table S-2. Summary of identified siloxane species.

Integer mass	Compound	Formula for M
149	$[M+H]^{+/-}$ $[M+H]^{+/-}$	$[C_2H_6SiO]_2$
223	$[M+H]^{+/-}$	$[C_2H_6SiO]_3$
297	$[M+H]^{+/-}$	$[C_2H_6SiO]_4$
355	$[M+H-CH_4]^+$	$[C_2H_6SiO]_5$
371	$[M+H]^{+/-}$	$[C_2H_6SiO]_5$
429	$[M+H-CH_4]^+$	$[C_2H_6SiO]_6$
445	$[M+H]^{+/-}$	$[C_2H_6SiO]_6$
462	$[M+NH_4]^+$	$[C_2H_6SiO]_6$
476	$[M+O_2]^-$	$[C_2H_6SiO]_6$
503	$[M+H-CH_4]^+$	$[C_2H_6SiO]_7$
519	$[M+H]^+$	$[C_2H_6SiO]_7$
536	$[M+NH_4]^+$	$[C_2H_6SiO]_7$
550	$[M+O_2]^-$	$[C_2H_6SiO]_7$
577	$[M+H-CH_4]^+$	$[C_2H_6SiO]_8$
593	$[M+H]^+$	$[C_2H_6SiO]_8$
610	$[M+NH_4]^+$	$[C_2H_6SiO]_8$
624	$[M+O_2]^-$	$[C_2H_6SiO]_8$
651	$[M+H-CH_4]^+$	$[C_2H_6SiO]_9$
667	$[M+H]^+$	$[C_2H_6SiO]_9$
684	$[M+NH_4]^+$	$[C_2H_6SiO]_9$
741	$[M+H]^+$	$[C_2H_6SiO]_{10}$

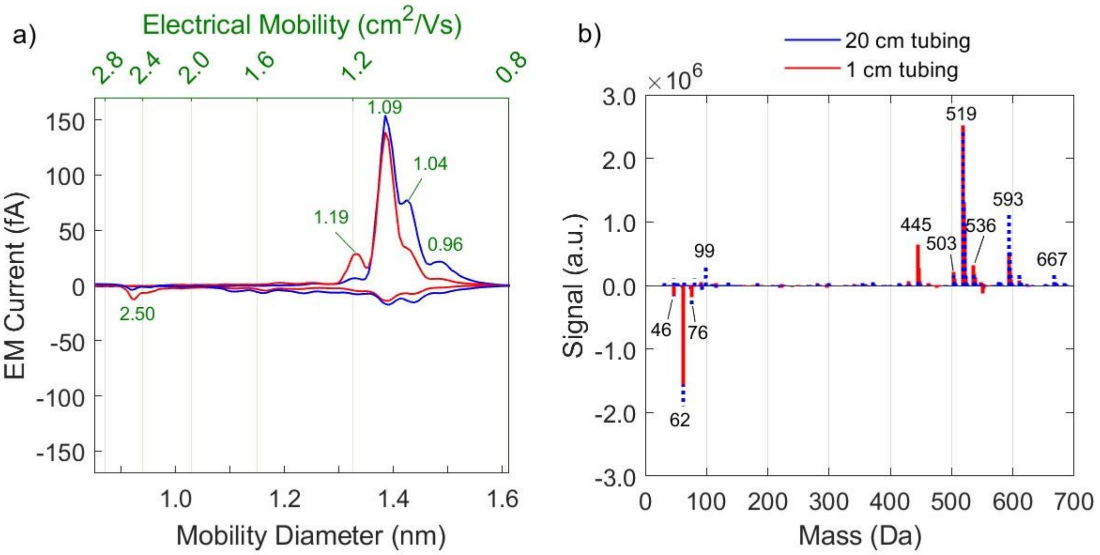

Figure S-3. Mobility (a) and mass (b) spectra of positive and negative ions produced by the RN in synthetic air, using a silicone tubing length of 20 cm and 1 cm upstream the charger.
